# From T cell engagers to next-generation immune cell engagers for cancer immunotherapy

**DOI:** 10.1093/abt/tbag030

**Published:** 2026-06-01

**Authors:** Zaopeng Yang, Yang-Xin Fu

**Affiliations:** Changping Laboratory, Beijing 102206, China; Changping Laboratory, Beijing 102206, China; Center for Cancer Biology, School of Basic Medical Sciences, Tsinghua University, Beijing 100084, China; State Key Laboratory of Molecular Oncology, Tsinghua University, Beijing 100084, China

**Keywords:** T cell engagers, immune cell engagers, γδ T cells, NK cell engagers, myeloid cell engagers, cancer immunotherapy

## Abstract

Immune cell engagers have emerged as a powerful class of multi-specific therapeutics that redirect immune effector cells toward tumor cells to induce targeted cytotoxicity. Among these, T cell engagers (TCEs) represent the most clinically advanced platform, with multiple approved agents demonstrating substantial efficacy in hematologic malignancies. However, their broader application remains limited by systemic toxicities, antigen heterogeneity, and reduced efficacy in solid tumors. To address these challenges, next-generation TCEs are being engineered with improved selectivity, and optimized signaling properties. In parallel, increasing attention has shifted toward engaging alternative immune effectors, including γδ T cells, natural killer cells, and myeloid populations, which provide complementary mechanisms of tumor recognition and immune modulation. In this Review, we summarize the biological principles underlying T or other immune cell engagers, highlight emerging alternative platforms, and discuss evolving engineering strategies that are shaping the future of programmable cancer immunotherapy.

## Teaser

Statement of Significance Immune cell engagers have rapidly evolved from T cell–redirecting antibodies into a diverse class of multi-specific therapeutics capable of mobilizing multiple immune cell populations. While T cell engagers (TCEs) have demonstrated remarkable clinical success, their limitations in solid tumors have prompted the exploration of alternative effector systems, including γδ T cells, natural killer cells, and myeloid cells. This Review summarizes the key features of currently approved TCEs and integrates emerging insights from next-generation immune cell engager platforms that are reshaping the design and therapeutic potential of future immune engagers.

## Introduction

Immunotherapy has transformed cancer treatment by restoring effective immune responses capable of controlling and eliminating tumors, demonstrating durable clinical benefit across multiple malignancies. Major immunotherapeutic strategies include adoptive cell therapies, immune checkpoint inhibitors, and targeted monoclonal antibodies. More recently, immune cell engagers have emerged as a distinct class of antibody-based therapeutics designed to redirect immune effector cells toward tumor cells. Most immune cell engagers consist of multi-specific antibody formats that simultaneously bind tumor-associated antigens (TAAs) and immune cell–surface molecules, thereby enforcing the formation of a functional immunological synapse between tumor and immune cells. Engagement of immune receptors triggers intracellular signaling cascades that activate cytotoxic machinery and promote targeted tumor cell killing [1].

**Figure 1:**
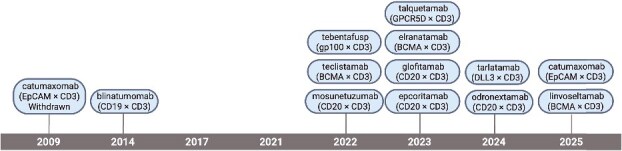
Timeline of TCEs approvals, representative T-cell engagers approved between 2009 and 2025, illustrating their target antigens and the evolution of T-cell redirecting therapies. Created with Biorender.com.

Among these modalities, T cell engagers (TCEs) have emerged as one of the most clinically successful platforms capable of redirecting cytotoxic immune responses toward malignant cells [2–4]. Reflecting this progress, 12 TCEs have been approved globally for oncology indications as end of 2025, with particularly strong efficacy demonstrated in hematological malignancies (Fig. 1). Despite these advances, several limitations remain that restrict broader application of TCEs. Although highly effective in hematologic cancers, their activity in solid tumors has been more limited. Major clinical challenges include cytokine release syndrome (CRS), post-treatment relapse, and insufficient efficacy in tumors characterized by antigen heterogeneity or immunosuppressive tumor microenvironments (TME) [5–7].

In response to these limitations, substantial efforts have focused on improving the design and performance of T cell engagers. These strategies include optimization of signaling strength, incorporation of co-stimulatory signaling modules. In parallel, increasing attention has shifted toward engaging alternative immune effector cell populations with intrinsic antitumor activity. Effector cells such as γδ T cells, natural killer (NK) cells, and myeloid cells possess unique recognition mechanisms that enable them to detect cellular stress or transformation independently of classical antigen presentation [1, 8]. Importantly, these cell types not only mediate direct cytotoxicity but also shape adaptive immune responses through antigen presentation, cytokine secretion, and immune orchestration.

## Approved TCEs as an emerging drug modality

The therapeutic concept of TCEs originated from early studies on bispecific antibodies (bsAbs). In 1985, CD3-targeting bsAbs were shown to redirect T cells to eliminate tumor cells independently of antigen presentation or co-stimulation, establishing the foundational principle of antibody-mediated T cell redirection [9]. This discovery established the core mechanistic principle of antibody-mediated T cell redirection that underlies modern TCE therapeutics. Subsequent engineering efforts focused on overcoming structural and manufacturing limitations. The introduction of single-chain variable fragments (scFvs) enabled compact [10], fully recombinant antibody formats, while later Immunoglobulin G (IgG)-engineering strategies, such as knobs-into-holes technology [11], markedly improved correct chain pairing, stability, and production efficiency. Despite these advances, TCEs remained largely preclinical until the approval of catumaxomab, a trifunctional αEpCAM × αCD3 TCE, in Europe in 2009 for malignant ascites [12], which provided the first clinical proof of concept for TCE immunotherapy.

The therapeutic potential of TCEs was firmly established with the FDA approval of blinatumomab, a αCD19 × αCD3 BiTE^®^, in 2014 for B-cell acute lymphoblastic leukemia [13], demonstrating that continuous engagement of endogenous T cells can induce durable remissions. Which demonstrated robust efficacy in acute lymphoblastic leukemia but required continuous infusion due to its short serum half-life [14–16]. To address these limitations, next-generation TCEs adopted Fc-silenced IgG backbones, achieving IgG-like pharmacokinetics. Following an eight-year gap after this milestone, the field has entered a phase of rapid expansion, with multiple agents approved by both the Food and Drug Administration (FDA) and European Medicines Agency (EMA) (Table 1). To date, 12 TCEs targeting seven antigens have been approved, including CD19, CD20, B-cell Maturation Antigen (BCMA), and GPRC5D in hematologic malignancies, as well as gp100–HLA, DLL3, and EpCAM in solid tumors. Among these, CD20-targeting TCEs such as mosunetuzumab, glofitamab, epcoritamab, and odronextamab have demonstrated strong efficacy in non-Hodgkin lymphoma, while BCMA-targeting agents including teclistamab, elranatamab, and linvoseltamab, together with the GPRC5D-targeting talquetamab, have reshaped the treatment landscape of multiple myeloma. In solid tumors, tebentafusp in uveal melanoma and tarlatamab in small cell lung cancer represent key clinical validations [34–36] (Table 2). Notably, all approved TCEs engage CD3 on T cells, although differences in epitope targeting, binding valency, and molecular architecture exist across agents (Table 1).

**Table 1:** Approved TCEs.

TCE	Indication	Dose and schedule	Route	Fc	MW	T_1/2_	CD3^+^ cell affinity	Target cell affinity	CD3/Target affinity ratio	References
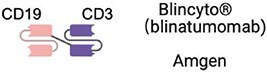	MRD^+^ or r/r or CP CD19^+^ B-ALL	MRD+, ≥45 kg bodyweight:four cycles of days 1–28, 28 μg/day coutinuous infusion; days 29–42, no treatment. Induction 29–42, no treatment. r/r: induction cycle 1, days 1–7, 9 μg/day; days 8–28, 28 μg/day; cycle 2, days 1–28, 28 μg/day; days cycles 3–5, days 1–28, 28 μg/day; days 29–42, no treatment. Cycles 6–9, days 1–28, 28 μg/day, days 29–84, CP: consolidation cycle, days 1–28, 28 μg/day; days 29–42, no treatment.	IV infusion	NA	54 kDa	2.1 h	260 nM	1.49 nM	1:175	[17–20]
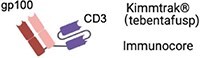	HLA-A*02:01 unresectable or metastatic UM	Weekly. Day 1, 20 μg; day 8, 30 μg ；then 68 μg weekly	IV infusion	NA	77 kDa	7.5 h	38 nM	24 pM	1: 1583	[18, 20–22]
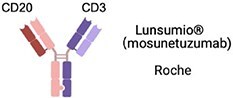	r/r FL	21 day cycles. Cycle 1 step-up, 1–60 mg; cycle 2, 60 mg, cycle 3+, 30 mg	IV infusion	humanized IgG1, attenuated Fc, N297G, aglycosylated, no Fc γR binding	146 kDa	6–11 d	40 nM	68 nM	1.7: 1	[20, 23]
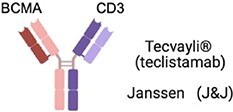	r/r MM	Weekly cycles. Cycle 1 step-up, 0.06–1.5 mg/kg; week 2+, 1.5 mg/kg. Patients with CR ≥ 6 m: 1.5 mg/kg every 2 wk	SC injection	humanized IgG4, L234A, L235A	147 kDa	15 d	28 nM	180 pM	1:156	[18, 20, 24]
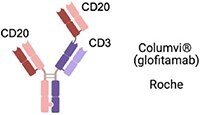	r/r DLBCL, NOS DLBCL, LBCL	21 day cycles. Cycle 1, day 1, obinutuzumab 1000 mg Pretreatment; day 8, 2.5 mg Columvi, day 15, 10 mg. Cycle 2–12, day 1, 30 mg	IV infusion	humanized lgG1, L234A, L235A, P329G LALAPG),no FcyR and Clqbinding	197 kDa	6-11d	>20 nM	4.8 nM	~1:4.2	[20, 25]
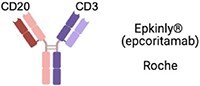	r/r DLBCL, r/r FL	28 day cycles. Cycle 1 step-up, 0.16–48 mg weekly. Cycles 2/3, 48 mg weekly. Cycles 4–9, 48 mg biweekly. Cycle 10+, 48 mg every 4 wk	IV infusion	humanized IgG1L234F, L235E,D265A (FEA), noFcyR and Clq binding	149 kDa	22 d	4.7 nM	2.4 nM	1: 2	[20, 26]
TCE	Indication	Dose and schedule	Route	Fc	MW	T_1/2_	CD3^+^ cell affinity	Target cell affinity	CD3/Target affinity ratio	References
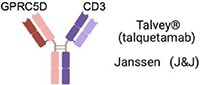	r/r MM	Two schedules. Weekly schedule: Cycle 1 step-up, day 1, 0.01 mg/kg; day 4, 0.06 mg/kg; day 7, 0.4 mg/kg. Then 0.4 mg/kg once weekly. Biweekly schedule: Cycle 1 as above. Day 10, 0.8 mg/kg; then 0.8 mg/kg every 2 wk.	SC injection	humanized IgG4 S228P, F234A L235A (IgG4-PAA minimized FcR binding	147 kDa	8.4–12.2 d	25 nM	9.7–14 nM	1:1.8–2.6	[20, 27, 28]
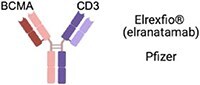	r/r MM	Two schedules. Weekly schedule: Cycle 1 step-up, day 1, 12 mg; day 4, 32 mg; day 8, 76 mg. Then 76 mg weekly through week 24. Biweekly schedule: responders only. Starting at week 25, 76 mg every 2 wk.	SC injection	humanized IgG2Δaκ, G2ΔA, D265A reduce FcγR binding	149 kDa	22 d	17 nM	20 pM	1: 425	[17, 29]
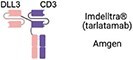	Pretreated ES-SCLC	Biweekly cycles. Cycle 1 step-up, day 1,1 mg; day8,10 mg; day15,10 mg Cycle 2+, days 1 and 15, 10 mg each .	IV infusion	IgG1-Fc with engineered CH2, N297G	105 kDa	11.2 d	14.9 nM	640 pM	1:23	[17, 30]
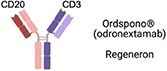	r/r DLBCL, r/r FL	Four 21-day cycles followed by biweekly or monthly maintenance.Cycle 1 step-up, day 1, 0.2 mg, day 2,0.5 mg, days 8 and 9, 2 mg, days 15 and 16, 10 mg. Cycles 2–4, 80 mg (r/rFL) or 160 mg (r/r DLBCL) on days 1,8 and 15. Maintenance, 160 mg (r/rFL) or 320 mg (r/r DLBCL) biweekly.Reduce to monthly after CR durationof 9 mo.	IV infusion	Effector function- minimized IgG4	150 kDa	ND	Not found	Not found	—	[20, 31]
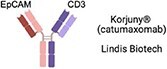	Malignant ascites in adults with EpCAM+ carcinomas	Four IP infusions of 10, 20, 50, and 150 μg on days 0, 3, 7, and 10, respectively.	IP infusion	rat/mouse hybrid，IgG2, quadroma, technology, FcγR binding intact	150 kDa	2.5 d	Not found	Not found	—	[12, 32]
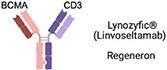	r/r MM	Step-up: three ascending doses of 5 mg on week 1, 25 mg on week 2, and 200 mg on week 3. Then 200 mg weekly in week 4–13. Patients who have received at least followed by 200 mg every 2 wk. 17 doses of 200 mg and shown confirmed VGPR or better at or after week 24 can switch to once every 4 wk.	IV infusion	human IgG4, Effector function- minimized IgG4	146 kDa	6.5–7.7 d in monkeys	120 nM	Not found	—	[17, 20, 33]

MW, molecular weight; NA, not applicable; T _1/2_, serum half-life; TAA, tumor-associated antigen; w, week(s); d, day(s); h, hour(s); r/r, relapsed or refractory.

**Table 2:** Clinical performance of approved TCEs.

**TCE**	**Indication**	**Efficacy**				**Safety**		**References**
		**ORR**	**mDOR**	**mPFS**	**mOS**	**CRS**	**TRSAE**	
Blinatumomab	r/r B-ALL, phase 3	CR 34% vs. 16%SOC		7.3 vs. 4.6 m SOC	7.7 vs. 4 m SOC	14.2% CRS	3% fatal, 4% CRS	[37]
	r/r B-ALL, real-world date	Ph-,46% CR, Ph+, 41% CR		Ph-,11 m, Ph+, 6.7 m	Ph-,12.2 m, Ph+, 16.3 m			[38]
Mosunetuzumab	r/r FL	80% ORR, 60% CR	22.8 m	17.9 m	89.6% (18 m)	43% CRS, 5% mild ICANS	47%	[23]
Epcoritamab	r/r LBCL, 2-year follow-up	53.1% ORR, 40.1% CR	17.3 m	4.4 m	18.5 m	51% CRS, 6.4% ICANS	11.5%, 0.6% fatal ICANS	[39]
	r/r FL	r/r FL, optimization cohort: 91% ORR, 68% CR				49% CRS, 0% ICANS		[40]
Glofitamab	r/r B-NHL	53.8% ORR, 36.8% CR (at RP2D: 65.7 and 57.1%)	5.5 m	2.9 m		50.3% CRS, 5.2% ICANS, 43.3%neurologic AE	45%, none fatal	[41]
	r/r MCL	85% ORR, 78.3% CR in small cohort	16.2 m	16.8 m	29.9 m	70% CRS, 11.7% neurologic AE	60%, none grade 5 (fatal)	[42]
Odronextamab	r/r FL	80% ORR,73% CR	22.6 m	20.7 m	NR	56% CRS, 0.8% ICANS	44.5%, 3% fatal	[31]
	r/r DLBCL	52% ORR, 31% CR	10.2 m			55% CRS, no ICANS	4% fatal COVID-19 infections	[31]
Teclistamab	r/r MM, 2-yeaar follow-up	Original ORR 63%, 58.8%VGPR or better; 2-year follow-p: 43% CR	24 m	12.5 m	21.9 m	72% CRS, 14.5% neurologic events, 3%ICANS	3.6% deaths	[24]
Elranatamab	r/r MM	Original, 56% VGPR or better, 35% CR; Prolonged follow-up: 61% ORR, 37.4% CR	NR	17.2 m	24.6 m	57.7% CRS, 4.9%ICANS	3.25% deaths (2.4% lethal infections)	[43]
Linvoseltamab	r/r MM	70% ORR, 45% CR (including 19% VGPR)	NR. Bumma 29.4 m	Bumma:NR	Bumma:31.4 m	46% CRS, 8% ICANS	74% SAE, 7% fatal	[44]
Talquetamab	r/r MM	73%–74% ORR, 57%–59% VGPR or better		7.5–11.9 m		75%–79% CRS, 11% ICANS	No deaths	[27]
Tebentafusp	Uveal melanoma	ORR 11% vs. 5% IC; CR < 1% vs. 0%	11.1 vs. 9.7 m	3.4 vs.2.9 m	21.6 vs. 16.9 m	89% CRS		[22]
Tarlatamab	Pretreated ES-SCLC	10 mg: 40% ORR, 1% CR. 100 mg: 32% ORR,8% CR		10 mg: 4.9 m. 100 mg: 3.9 m	10 mg: 14.3 m. 100 mg: NE	10 mg: 51% CRS，8% ICANS. 100 mg: 61% CRS, 28% ICANS	10 mg: 1% fatal respiratory failure	[20]
Catumaxomab	Malignant ascites inadults with EpCAM+ carcinomas				72 vs. 71d (control)	23% CRS	15%. Integrated analysis of 11 studies: SIRS, hepatic failure	[20, 32]

Data are from published pivotal trials, including recent updates and select real-world data. ORR, objective response rate; mDOR:median duration of response; mPFS, median progression-free survival; OS, overall survival (duration in parentheses); TRSAE, treatment-related serious adverse events; NE, not evaluated; NR, not reached; Ph, Philadelphia chromosome; r/r, relapsed or refractory; RP2D, recommended phase 2 dose; SIRS, systemic inflammatory response syndrome; VGPR, very good partial response.

### Mode of action and design principles

Mechanistically, TCEs function as molecular adaptors that bridge T cells and target cells through simultaneous binding to a TAA and CD3 within the T cell receptor (TCR) complex [45–47]. Among the TCR subunits, CD3ε is most commonly targeted, enabling TCEs to bypass peptide–MHC (pMHC) recognition and directly induce the formation of a cytolytic immune synapse [48–50]. This interaction triggers canonical TCR signaling cascades, including Lck-mediated phosphorylation of CD3 ITAMs, exclusion of CD45, and activation of ZAP-70, ultimately propagating downstream signaling via Linker for Activation of T cells (LAT) and SLP-76 [51] . Functionally, this leads to T cell activation, cytokine release, proliferation, and perforin/granzyme-mediated cytotoxicity [52, 53]. A defining feature of this mechanism is its high signaling efficiency, enabling potent cytotoxicity at picomolar concentrations [54, 55]. Clinically, this is exemplified by agents such as teclistamab [56] and elranatamab [57], which induce deep responses in heavily pretreated multiple myeloma. This efficiency likely reflects high antigen density and the formation of TCR-enriched immune synapses that support multi-T cell engagement and serial killing.

Despite this seemingly straightforward mechanism, TCE activity is strongly influenced by molecular architecture and biophysical parameters, including antigen density, epitope location, binding affinity, and intercellular spacing [58]. These principles are reflected in clinically successful formats such as the 2:1 configuration of glofitamab and the monovalent CD3 engagement of epcoritamab [26]. Notably, the broad success of structurally diverse TCEs suggests a high degree of tolerance in synapse geometry, and optimal design remains largely empirical. A key design requirement that T cells are triggered only in the presence of target cells. This is typically achieved through monovalent CD3 binding, affinity tuning, and Fc silencing to minimize off-target activation [59, 60]. From a translational perspective, the high potency of TCEs necessitates careful dose selection: T cell–dependent cytotoxicity can be triggered at extremely low concentrations, and even a small number of engaged receptors may suffice for full activation [54, 61] (Table 1).

### Limitations in solid tumors

Despite these advances, the translation of TCEs into solid tumors remains limited. Unlike hematologic malignancies, solid tumors often lack co-stimulatory signals, exhibit heterogeneous antigen expression, and are embedded within an immunosuppressive TME characterized by hypoxia, metabolic competition, and inhibitory cytokines [7, 62]. These factors collectively limit T-cell infiltration, activation, and persistence. In addition to suboptimal therapeutic activity, TCE therapy is often associated with a narrow therapeutic index and significant treatment-related toxicities, most notably cytokine release syndrome (CRS) and immune effector cell–associated neurotoxicity syndrome (ICANS), which further hinder their safe and effective application in solid tumors.

Although agents such as tebentafusp and tarlatamab demonstrate that TCEs can achieve clinical benefit in selected solid tumors, these successes often depend on unique biological contexts rather than broadly applicable principles. For example, tebentafusp leverages peptide–MHC–restricted targeting to access intracellular antigens, while tarlatamab targets DLL3, a lineage-associated antigen with relatively homogeneous expression and tumor-restricted signaling dependencies. Notably, DLL3 has been implicated in the modulation of Notch signaling [63] and may contribute to an immune-permissive context through mechanisms such as PD-L1 upregulation [64], potentially shaping local tumor–immune interactions. In addition, such settings may partially compensate for the lack of classical co-stimulatory signals through intrinsic tumor biology or a more permissive immune microenvironment. These constraints highlight that signal-1–driven T cell redirection alone is often insufficient to achieve durable responses in solid tumors. Effective TCE therapy in this setting will likely require additional layers of regulation to enhance T cell function in a spatially and temporally controlled manner, thereby motivating the development of next-generation, functionally enhanced TCEs that integrate complementary signals beyond CD3 activation.

## TCEs with co-stimulatory signaling

Tumor-targeting co-stimulatory bsAbs provide a conditional “signal 2” to enhance T cell activation by bridging a TAA with co-stimulatory receptors such as 4-1BB (CD137) or CD28 on T cells, promoting receptor clustering in trans at the tumor-immune synapse [65, 66]. Among these pathways, 4-1BB has been the most extensively exploited. Its inducible expression following T cell activation and central role in sustaining effector function, and survival have made it an attractive target. Contemporary 4-1BB targeting bsAbs are no longer designed as systemically active agonists, but instead rely on tumor-restricted receptor clustering to uncouple efficacy from hepatotoxicity observed with first-generation agonistic antibodies. Two main design paradigms have emerged. One utilizes active agonistic anti-4-1BB antibodies enabled by tumor antigen engagement, exemplified by PRS-343 (cinrebafusp), an αHER2 × α4-1BB anticalin-IgG fusion demonstrating favorable tolerability and on-tumor immune activation [67]. The second employs membrane-anchored 4-1BB ligand (4-1BBL) mimetics to achieve more physiological receptor clustering, as illustrated by FAP-4-1BBL (RG7872), which demonstrated immune activation as monotherapy and enhanced efficacy in combination with PD-L1 blockade [68]. Collectively, these agents highlight how diverse tumor antigens, including Epidermal Growth Factor Receptor (EGFR), PDL1, Prostate-Specific Membrane Antigen (PSMA), CLDN18.2, B7-H4, CEACAM5, HER2, and Fibroblast Activation Protein (FAP), can be leveraged to spatially constrain 4-1BB co-stimulation, enabling potent yet context-dependent T cell activation within the TME [65].

CD28 represents an alternative, non-redundant co-stimulatory axis exploited in tumor-targeted bsAb designs. Constitutively expressed on naïve and memory T cells, CD28 provides a potent early activation signal through engagement with CD80 or CD86 on professional antigen-presenting cells [69]. However, the catastrophic cytokine release syndrome observed with the CD28 super agonist TGN1412 underscored the extreme sensitivity of this pathway to systemic activation and emphasized the necessity of strict contextual control [70]. Tumor-targeted CD28 bsAbs offer such control by confining CD28 engagement to antigen-positive tumor sites [71]. In contrast to 4-1BB, which predominantly sustains late-stage effector function and persistence, CD28 primarily amplifies early T cell activation and proliferation. This functional distinction suggests that CD28-based co-stimulatory TCEs may be particularly effective in priming weak or subthreshold TCR signals, whereas 4-1BB-based approaches are better suited to reinforcing ongoing effector responses.

Despite these advances, emerging clinical data indicate that the translation of co-stimulation remains constrained by several practical challenges. Second-generation agonists generally exhibit favorable safety profiles, with most adverse events limited to grade 1–2 immune-related toxicities, such as rash, pruritus, pneumonitis, or transient liver enzyme elevations [72]. While these immune-related adverse events (irAEs) may correlate with improved clinical outcomes in some settings [73], their management remains critical for maintaining therapeutic benefit. More importantly, optimal dosing and scheduling strategies are still not well defined. Clinical studies have explored a wide range of regimens, including weekly to every 3–6 weeks administrations, using both flat and weight-based dosing approaches. Pharmacodynamic readouts, such as induction of IFNγ, CXCL10, Ki67^+^ CD8^+^ T cells, and NK cell activation, suggest that biological activity can be achieved across a relatively broad dose range, yet the therapeutic window remains incompletely characterized [74].

Several clinical strategies now pair co-stimulatory bsAbs with CD3-TCEs. For example, REGN5837, a αCD22 × αCD28 bsAb currently evaluated in combination with the αCD20 × αCD3 TCE odronextamab for non-Hodgkin lymphoma [75] and Englumafusp alfa (αCD19 × 4-1BBL) is currently evaluated in combination with the αCD20 × αCD3 TCE glofitamab in diffuse large B cell lymphoma [76]. These studies highlight how tumor-targeted co-stimulatory signals can be harnessed to reinforce CD3-mediated activation. Building on this principle, tri-specific TCEs integrate CD3 engagement and co-stimulatory signaling within a single molecule, offering coordinated delivery and potentially simpler clinical implementation. CD28-containing tri-specifics, such as αHER2 × αCD3 × αCD28 (SAR443216) and αCD38 × αCD3 × αCD28 (SAR442257), have demonstrated robust T cell activation and cytotoxicity in early clinical development, even against tumors with low antigen density [77–79]. Tri-specific formats incorporating 4-1BB or CD28 signaling, including αPSMA × αCD3-based constructs [80], aim to reinforce effector durability and metabolic fitness following initial TCR engagement, clinical plan may be easier for tri-specific, although there may be less control on acute toxicity.

Beyond these classical co-stimulatory pathways, the CD2-CD58 axis has emerged as a complementary and mechanistically distinct co-stimulatory module that is particularly well suited for integration into tri-specific TCE formats. CD2 is broadly expressed across naïve, effector, and exhausted T cell subsets and contributes to immune synapse stabilization, signal amplification, and sustained T cell-target cell adhesion through interaction with CD58 [81, 82]. Unlike CD28 or 4-1BB, CD2 signaling primarily enhances the quality and persistence of TCR signaling rather than acting as a strong independent activation trigger [83], making it especially compatible with CD3-based redirection strategies. Preclinical and clinical observations indicate that intact CD58 expression on tumor cells augments TCE potency, whereas CD58 loss confers resistance to CD3-based TCE therapies, underscoring the functional relevance of this pathway in T cell-mediated tumor control. Accordingly, tri-specific constructs that integrate tumor antigen recognition, CD3 engagement, and CD2 co-stimulation, such as emerging αCD3 × αTAA × αCD2 platforms [84], aim to stabilize immunological synapse formation and sustain T cell activity under conditions of low antigen density or impaired classical co-stimulation.

Collectively, co-stimulatory and tri-specific TCEs represent a rational evolution of T cell-redirecting therapies. By integrating signal-1 with complementary co-stimulatory modules, CD28 and 4-1BB to amplify activation magnitude and CD2 to stabilize signaling durability, these platforms offer a flexible framework to tailor T cell responses to the complex immunobiology of solid tumors. Beyond signal-2, the conditional incorporation of signal-3 cytokine cues may further enhance T cell persistence and function, provided cytokine-associated toxicities can be appropriately controlled.

## γδ T cell engagers

Conventional αβ T cells rely on pMHC complexes for antigen recognition, whereas γδ T cells can recognize and eliminate transformed cells in an MHC-independent manner through sensing stress-induced ligands that are frequently upregulated in malignant tissues [85]. This unique recognition mode confers γδ T cells with the potential for broad, population-wide immunotherapy that is less constrained by HLA diversity [86]. In addition, γδ T cells recognize multiple stress-associated antigens that are commonly shared across malignancies regardless of tissue origin, further supporting their suitability as universal immune effectors [87].

Functionally, γδ T cells can mediate tumor cell killing through cytotoxic mechanisms including perforin/granzyme release and Fc receptor–mediated antibody-dependent cellular cytotoxicity (ADCC). For example, modular γδ-TCE systems targeting hematologic malignancies such as CD33 in AML and CD19 [88, 89] in B-cell acute lymphoblastic leukemia (B-ALL) have demonstrated the ability to promote activation and expansion of both Vδ1^+^ and Vδ2^+^ γδ T cell populations. Such platforms are proposed to support sustained expansion of diverse γδ T cell repertoires, thereby maintaining persistent antitumor pressure within the TME. In preclinical settings, these constructs enhanced γδ T cell–mediated cytotoxicity against antigen-positive tumor cells in a dose-dependent manner and promoted increased degranulation and secretion of inflammatory cytokines, including IFN-γ, TNF-α, granzymes, and perforin [90].

**Figure 2:**
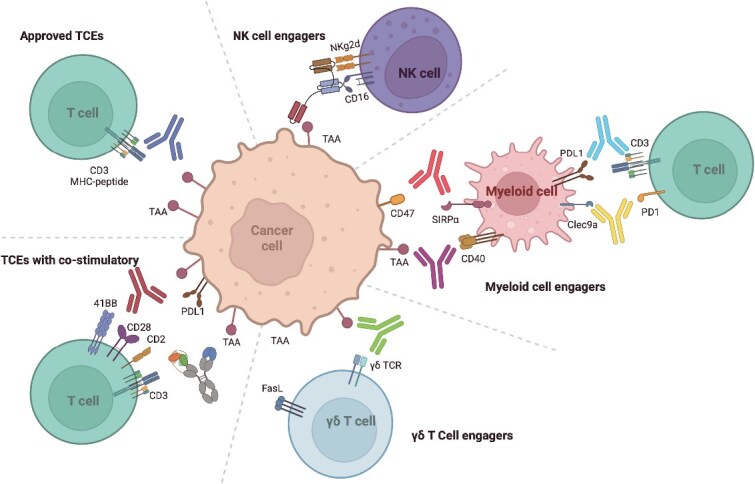
Mode of action of immune cell engagers. Created with Biorender.com.

Building on this biology, γδCEs (Fig. 2) have been engineered to selectively recruit γδ T cells to tumor targets while leveraging their innate-like recognition capabilities. One common design strategy involves bispecific constructs composed of Vγ9 TCR and scFvs targeting TAAs such as HER2 [91] or CD123 [92]. These molecules have demonstrated efficient tumor cell killing in vitro and robust activity in preclinical models. Alternative strategies employ bispecific antibodies that directly target the Vδ2 TCR chain together with TAAs using VHH-based formats. Such constructs have been developed against targets including CD40, CD1d, which is expressed in AML, chronic lymphocytic leukemia (CLL) [93, 94], and EGFR, which is frequently overexpressed in epithelial cancers [95]. These approaches expand the range of tumor indications potentially addressable by γδCE-based therapies.

Clinical translation of γδCEs is currently underway. For example, PF-08046052 (also known as SGN-EGFRd2，NCT05983133) represents an investigational bispecific γδ T cell engager directed against EGFR. This molecule consists of a humanized heavy chain–only bispecific antibody designed to conditionally activate Vγ9Vδ2 T cells through simultaneous binding to EGFR and the γδ TCR. PF-08046052 is currently being evaluated in early-phase clinical trials in patients with advanced solid tumors.

## Beyond T cell engagers

### NK cell engagers

NK cells are potent innate lymphocytes capable of directly killing tumor cells, including those with stem-like features, and play important roles in controlling hematological malignancies such as leukemia and lymphoma [96, 97]. Their antitumor activity can be therapeutically harnessed either through infusion of activated or engineered NK cells, or indirectly via cytotoxic monoclonal antibodies that induce ADCC. The approval of Rituximab marked the first approved cytotoxic mAb, followed by over 30 others targeting antigens like EGFR and HER2, often engineered to enhance Fc binding to CD16A and boost NK-mediated responses [98].

Building on these principles, NK cell engagers (NKCEs) (Fig. 2) have emerged as an innovative class of immune-therapeutics that redirect endogenous NK cells to tumor targets. By simultaneously binding TAAs, such as HER2, CD19, or BCMA and activating NK receptors, NKCEs promote targeted cytotoxicity while generally maintaining a favorable safety profile compared with CD3-based T cell engagers, including a lower incidence of cytokine release syndrome [1]. Mechanistically, NKCE-mediated activation relies on engagement of NK activating receptors that regulate immune synapse formation and cytotoxic function. NK cell activity is governed by a dynamic balance between activating and inhibitory signals that maintain immune homeostasis and prevent unintended tissue damage. Key activating receptors include Fcγ receptor CD16A (FcγRIII), C-type lectin receptors such as CD94/NKG2C and NKG2D, and natural cytotoxicity receptors including NKp30 and NKp46 [8]. Engagement of these receptors facilitates immune synapse formation, leading to perforin- and granzyme-mediated cytotoxicity as well as cytokine secretion.

NKCEs have been developed in a range of multi-specific molecular formats designed to optimize receptor engagement and activation strength. Bispecific NKCEs typically bridge a single NK receptor, most commonly CD16Ato a tumor antigen [99, 100]. Tri-specific designs add a second NK-activating receptor (e.g. NKp46 or NKG2D), enabling synergistic signaling that amplifies activation beyond single-receptor engagement [1, 100–102], classic examples include TriKEs (trispecific killer engagers), which incorporate an IL-15 moiety to drive NK cell proliferation, persistence, and self-sustaining expansion without exogenous cytokine support [103]. More advanced tetra-specific formats further co-engage multiple receptors simultaneously while integrating engineered cytokine elements, such as non-α IL-2 variants (IL-2v). This modification selectively stimulates intermediate-affinity IL-2Rβ/γ complexes expressed on NK cells [104]. Collectively, those multi-receptor co-engagement strategies and targeted cytokine delivery approaches aim to promote sustained NK cell activation and superior cytotoxicity, particularly against antigen-low or therapy-resistant tumors, overcoming limitations of conventional monoclonal antibodies.

Conceptually, NK cell engagers address several intrinsic limitations of CD3-based TCEs. Unlike T cells, NK cells do not require antigen-specific priming or clonal expansion, and their activation thresholds are governed by the balance of activating and inhibitory signals rather than strict TCR-pMHC recognition. This biology may render NKCEs less dependent on high target antigen density and potentially less cytokine release. Moreover, NK cells remain functional in settings of T cell exhaustion or profound lymphodepletion, conditions frequently encountered in heavily pretreated patients. However, NK cell activity is highly sensitive to suppressive cues within the TME, including TGF-β, and hypoxia. Thus, NKCEs should be viewed not as direct replacements for TCEs, but as complementary modalities whose strengths may be best realized in specific biological or clinical contexts.

### Myeloid cell engagers

Unlike NK cells, whose antitumor activity is primarily mediated through direct cytolysis, myeloid cells-including dendritic cells, macrophages, and monocytes, serve as central regulators of antigen presentation, co-stimulation, and immune orchestration within the TME [98, 105]. Consequently, myeloid cell engagers (MCEs) (Fig. 2) are conceptually positioned not as immediate cytotoxic agents, but as modulators of immune priming and intratumoral immune architecture.

Among myeloid-associated targets, CD40 has emerged as a prototypical entry point for MCE development. Agonistic CD40 signaling promotes APC maturation, enhances antigen cross-presentation, and supports robust CD8^+^ T cell priming; however, systemic administration of CD40 agonistic monoclonal antibodies has been hampered by dose-limiting toxicities [106]. To address this challenge, tumor-targeted bsAbs that couple CD40 engagement with TAAs aim to spatially restrict receptor clustering to the TME, thereby decoupling immune activation from systemic inflammation. Preclinical studies of αCD40 × αCEACAM5, αCD40 × αEpCAM, and αmesothelin × αCD40 formats [107, 108] have demonstrated enhanced APC activation and secondary T cell responses with improved safety profiles compared with monospecific CD40 agonists.

A major focus of antibody-dependent cellular phagocytosis (ADCP) strategies involves targeting phagocytosis checkpoint pathways, particularly the SIRPα–CD47 axis, in which tumor cells express CD47 to deliver a “do not-eat-me” signal that suppresses macrophage activity. Blocking this interaction restores macrophage-mediated tumor clearance [109]. For example, the bispecific antibody HMBD004, targeting CD47 and CD33, demonstrated potent antitumor activity in preclinical models of AML [110]. Similar macrophage-engaging antibodies that simultaneously target TAAs while blocking SIRPα signaling have been shown to significantly enhance macrophage phagocytic activity and stimulate downstream CD8^+^ T cell–mediated tumor killing in murine tumor models, highlighting their capacity to bridge innate and adaptive immune responses [111].

Beyond macrophage activation, MCE strategies increasingly aim to restore productive myeloid–lymphoid crosstalk that is often defective in solid tumors. Dendritic cell-T cell co-engagers represent an emerging subclass designed to physically connect antigen-presenting cells with effector T cells. For example，bispecific *αPD-1 × αCLEC9A* constructs link PD-1^+^ T cells with cDC1 dendritic cells, promoting enhanced antigen presentation and superior immune reprogramming compared with conventional checkpoint blockade in preclinical models [112]. Similarly, αCD3 × αPD-L1 bispecific antibodies redirect T cell interactions toward PD-L1 expressing dendritic cells, integrating checkpoint inhibition with localized co-stimulation and resulting in more durable antitumor immunity [113]. Additional formats further expand this concept. A bispecific dendritic cell–T cell engager (BiDT), consisting of an anti-TIM3–IFN fusion protein, has been shown in preclinical tumor models to simultaneously target TIM3 on exhausted tumor-infiltrating lymphocytes (TILs) while activating dendritic cells through IFNAR signaling, thereby enhancing antigen presentation and reversing T cell dysfunction [114].

In parallel, tumor-associated macrophages (TAMs), particularly TREM2 expressing immunosuppressive subsets, have emerged as attractive targets for MCE-based immune remodeling. Myeloid-targeted IL-2 engagers that combine TREM2 blockade with protease-activated IL-2 achieve tumor-restricted activation of cytotoxic lymphocytes and induce coordinated remodeling of both innate and adaptive immune compartments [115]. Beyond cytokine-based approaches, macrophage-T cell engagers have also been developed to selectively eliminate immunosuppressive TAM populations. For example, Scott et al. engineered bi-valent T cell engagers fusing CD3 scFv targeting T cells and CD206 VHH or folate receptor b scFv targeting M2-like TAMs. They further developed tri-valent T cell engagers, incorporating an additional anti-CD3 or anti-CD28 domain to enhance potency [116]. Together, these approaches underscore the potential of MCEs to overcome defective priming, disrupted cellular communication, and dominant myeloid-driven immunosuppression characteristic of solid tumors.

Importantly, despite their compelling biological rationale, MCEs remain at an early stage of development. To date, the majority of reported MCE platforms are supported predominantly by preclinical proof-of-concept studies, with limited clinical validation. Key challenges include myeloid cell heterogeneity, dynamic target expression across tumor types and disease stages, and the risk of excessive or misdirected innate immune activation. As such, MCEs are unlikely to function as standalone therapies, but may instead find their greatest utility as immune “orchestrators” that synergize with T cell or NK cell directed engagers by reinstating effective antigen presentation and co-stimulation within the TME.

## Future perspectives

Across TCEs, preclinical and clinical experience converges on a central principle: while immune redirection can drive potent antitumor activity, unrestrained systemic activation remains a major barrier to efficacy and safety (Table 2), particularly in solid tumors, due to the paucity of truly tumor-specific antigens and the risk of on-target off-tumor toxicity, as many epithelial TAAs are also expressed in normal tissues [117]. Consequently, next-generation TCEs design is shifting from maximizing intrinsic potency toward engineering conditional, spatially restricted immune activation.

To address this, TCEs have been engineered as functional prodrugs that remain inert in circulation and are selectively activated within the tumor microenvironment [118]. Protease-cleavable steric masking represents the most advanced strategy, giving rise to protease-activated TCEs (probodies) that exploit elevated tumor-associated protease activity to confine CD3 engagement and reduce systemic toxicity [46, 52, 119, 120]. Alternatively, tumor-dependent assembly strategies, such as hemibody designs, split CD3-binding domains across inactive components that reconstitute activity only upon co-localized tumor antigen binding [121], introducing additional spatial logic to further limit off-tumor T cell activation.

Complementary to molecular conditionality, local and in situ delivery strategies seek to achieve high intratumoral TCEs concentrations while minimizing systemic exposure. Approaches under investigation include lipid nanoparticle mediated mRNA delivery encoding TCEs, exemplified by a CLDN6-targeted TCE [122, 123], as well as oncolytic virus platforms engineered for intratumoral TCE expression [124, 125]. Gene therapy like strategies further extend this concept, such as engineered CAR-T cells designed to locally secrete TCEs, effectively converting immune cells into intratumoral TCE factories [126, 127]. These delivery modalities provide an orthogonal solution to pharmacokinetic and safety limitations inherent to systemic TCE administration and underscore spatial confinement as a unifying design principle across emerging immune engager platforms.

Beyond improvements in biological design and delivery, the engineering platforms used to construct immune cell engagers are themselves rapidly evolving. Traditionally, most engager molecules have been generated through recombinant genetic fusion strategies. However, alternative engineering approaches are increasingly expanding the design landscape.

Nanomedicine-based assemblies, for example, utilize supramolecular chemistry or nanoparticle scaffolds to present multiple binding ligands on a single platform. Owing to their high surface-area-to-volume ratio, these systems enable multivalent and multi-specific immune engagement while offering additional opportunities for controlled release and modular customization [128, 129]. Such nanoparticle-based architectures may also alleviate challenges associated with recombinant protein expression, including aggregation or misfolding.

In parallel, chemical conjugation strategies have emerged as highly modular platforms for constructing immune co-engagers. These approaches employ bio-orthogonal reactions or enzymatic ligation methods to covalently assemble distinct functional components, including full-length antibodies, antigen-binding fragments, and immunomodulatory payloads into defined multi-specific architectures [129–131]. Compared with conventional genetic fusion, chemical assembly provides greater flexibility in combining diverse modules and allows rapid generation of structurally heterogeneous engager formats.

Collectively, these evolving strategies highlight a fundamental shift in immune engager development—from static protein engineering toward dynamic, modular, and spatially programmable systems. Continued innovation across molecular design, delivery technologies, and assembly platforms will likely be essential for overcoming the remaining barriers to safe and effective immune redirection, particularly in the context of solid tumors.

## Data Availability

No new data were generated or analyzed in support of this review.
